# Association of *LRRK2* p.A419V with Parkinson’s Disease in East Asians and analysis of age at onset

**DOI:** 10.1038/s41531-026-01265-3

**Published:** 2026-02-02

**Authors:** Kai Shi Lim, Maria Teresa Periñan, Elaine Guo Yan Chew, Paul Suhwan Lee, Fulya Akçimen, Jia Lun Lim, Mathew J. Koretsky, Manabu Funayama, Hiroyo Yoshino, Nobutaka Hattori, Rauan Kaiyrzhanov, Henry Houlden, Mariam Isayan, Yi Wen Tay, Tzi Shin Toh, Lei-Cheng Lit, Anis Nadhirah Khairul Anuar, Hans Xing Ding, Laurel Screven, Norlinah Mohamed Ibrahim, Chin-Hsien Lin, Han-Joon Kim, Jee-Young Lee, Sun Ju Chung, Jia Nee Foo, Eng-King Tan, Shen-Yang Lim, Ai Huey Tan, Sara Bandres-Ciga, Azlina Ahmad-Annuar, the Global Parkinson’s Genetics Program (GP2), Kai Shi Lim, Maria Teresa Periñan, Paul Suhwan Lee, Fulya Akçimen, Jia Lun Lim, Mathew J. Koretsky, Manabu Funayama, Hiroyo Yoshino, Nobutaka Hattori, Rauan Kaiyrzhanov, Henry Houlden, Mariam Isayan, Yi Wen Tay, Tzi Shin Toh, Lei-Cheng Lit, Hans Xing Ding, Laurel Screven, Norlinah Mohamed Ibrahim, Chin-Hsien Lin, Han-Joon Kim, Jee-Young Lee, Sun Ju Chung, Jia Nee Foo, Eng-King Tan, Shen-Yang Lim, Ai Huey Tan, Sara Bandres-Ciga, Azlina Ahmad-Annuar, Elaine Guo Yan Chew, Anis Nadhirah Khairul Anuar, Mie Rizig, Njideka Okubadejo, Emilia M. Gatto, Marcelo Kauffman, Samson Khachatryan, Zaruhi Tavadyan, Claire E. Shepherd, Julie Hunter, Kishore Kumar, Melina Ellis, Miguel E. Rentería, Sulev Koks, Alexander Zimprich, Carlos Rieder, Vitor Tumas, Sarah Camargos, Edward A. Fon, Ted Fon, Oury Monchi, Benjamin Pizarro Galleguillos, Patricio Olguin, Marcelo Miranda, Maria Leonor Bustamante, Beisha Tang, Huifang Shang, Jifeng Guo, Piu Chan, Wei Luo, Gonzalo Arboleda, Jorge Orozco, Marlene Jimenez del Rio, Alvaro Hernandez, Mohamed Salama, Walaa A. Kamel, Yared Z. Zewde, Alexis Brice, Jean-Christophe Corvol, Ana Westenberger, Christine Klein, Eva-Juliane Vollstedt, Harutyun Madoev, Joanne Trinh, Johanna Junker, Katja Lohmann, Anastasia Illarionova, Brit Mollenhauer, Franziska Hopfner, Günter Höglinger, Lara M. Lange, Manu Sharma, Thomas Gasser, Zih-Hua Fang, Sergio Groppa, Albert Akpalu, Georgia Xiromerisiou, Georgios Hadjigeorgiou, Efthymios Dardiotis, Ioannis Dagklis, Ioannis Tarnanas, Leonidas Stefanis, Maria Stamelou, Alex Medina, Germaine Hiu-Fai Chan, Nelson Yuk-Fai Cheung, Nancy Ip, Phillip Chan, Xiaopu Zhou, Asha Kishore, K. P. Divya, Pramod Pal, Prashanth Lingappa Kukkle, Roopa Rajan, Rupam Borgohain, Mehri Salari, Andrea Quattrone, Enza Maria Valente, Micol Avenali, Lucilla Parnetti, Tommaso Schirinzi, Tomotaka Shiraishi, Altynay Karimova, Gulnaz Kaishibayeva, Cholpon Shambetova, Rejko Krüger, Nor Azian Abdul Murad, Shahrul Azmin, Wael Mohamed, Daniel Martinez-Ramirez, Mayela Rodriguez-Violante, Paula Reayes-Perez, Bayasgalan Tserensodnom, Rajeev Ojha, Tim J. Anderson, Toni L. Pitcher, Oluwadamilola Ojo, Jan O. Aasly, Lasse Pihlstrøm, Manuela Tan, Shoaib Ur-Rehman, Mario Cornejo-Olivas, Maria Leila Doquenia, Raymond Rosales, Angel Vinuela, Elena Iakovenko, Bashayer Al Mubarak, Muhammad Umair, Ferzana Amod, Jonathan Carr, Soraya Bardien, Beomseok Jeon, Yun Joong Kim, Esther Cubo, Ignacio Alvarez, Janet Hoenicka, Katrin Beyer, Pau Pastor, Sarah El-Sadig, Christiane Zweier, Paul Krack, Ruey-Meei Wu, Hsiu-Chuan Wu, Yih-Ru Wu, Pin-Jui Kung, Serena Wu, Rim Amouri, Samia Ben Sassi, A. Nazl Başak, Özgür Öztop Çakmak, Sibel Ertan, Gencer Genc, Alejandro Martínez-Carrasco, Anette Schrag, Anthony Schapira, Eleanor J. Stafford, Huw Morris, John Hardy, Nicholas Wood, Olaitan Okunoye, Rimona Weil, Simona Jasaitye, Vida Obese, Camille Carroll, Claire Bale, Donald Grosset, Kin Y. Mok, Nigel Williams, Patrick A. Lewis, Seth Love, Simon Stott, Alberto Espay, Luca Marsili, Alyssa O’Grady, Bernadette Siddiqi, Bradford Casey, Brian Fiske, Charisse Comart, Justin C. Solle, Kaileigh Murphy, Maggie Kuhl, Naomi Louie, Sohini Chowdhury, Todd Sherer, Andrew K. Sobering, Cabell Jonas, Carlos Cruchaga, Caroline B. Pantazis, Claire Wegel, Deborah Hall, Ejaz Shamim, Jared Williamson, Ekemini Riley, Sonya Dumanis, Geidy E. Serrano, Thomas Beach, Honglei Chen, Ignacio Juan Keller Sarmiento, Niccolò E. Mencacci, Steven Lubbe, Joseph Jankovic, Miguel Inca-Martinez, Joshua Shulman, Karen Nuytemans, Karl Kieburtz, Katerina Markopoulou, Kenneth Marek, Lana M. Chahine, Lauren Ruffrage, Marissa Dean, Lisa Shulman, Roger Albin, Roy Alcalay, Ruth Walker, Tao Xie, Tatiana Foroud, Duan Nguyen, Toan Nguyen, Masharip Atadzhanov

**Affiliations:** 1 Department of Biomedical Science, Faculty of Medicine, Universiti Malaya, 50603 Kuala Lumpur, Malaysia; 2 Department of Medicine, Faculty of Medicine, Universiti Malaya, 50603 Kuala Lumpur, Malaysia; 3 Unidad de Trastornos del Movimiento, Servicio de Neurología y Neurofisiología Clínica, Instituto de Biomedicina de Sevilla, Hospital Universitario Virgen del Rocío, Consejo Superior de Investigaciones Científicas (CSIC), Universidad de Sevilla, Seville, Spain; 4 Centre for Preventive Neurology, Wolfson Institute of Population Health, Queen Mary University of London, London, United Kingdom; 5 Lee Kong Chian School of Medicine, Nanyang Technological University Singapore, Singapore, Singapore; 6 Center for Alzheimer’s and Related Dementias, National Institute on Aging and National Institute of Neurological Disorders and Stroke, National Institutes of Health, Bethesda, Maryland USA; 7 Laboratory of Neurogenetics, National Institute on Aging, National Institutes of Health, Bethesda, MD USA; 8 The Mah Pooi Soo & Tan Chin Nam Centre for Parkinson’s & Related Disorders, Faculty of Medicine, Universiti Malaya, Kuala Lumpur, Malaysia; 9 Department of Neurology, Faculty of Medicine, Juntendo University, 2-1-1 Hongo, Bunkyo-ku, Tokyo, 113-8421 Japan; 10 Research Institute for Diseases of Old Age, Graduate School of Medicine, Juntendo University, 2-1-1 Hongo, Bunkyo-ku, Tokyo, 113-8421 Japan; 11 International Collaborative Research Administration, Juntendo University, Tokyo, Japan; 12 Department of Neuromuscular Diseases, UCL Queen Square Institute of Neurology, University College London, London, UK; 13 South Kazakhstan Medical Academy, Department of Neurology, 1/1 Al-Farabi Avenue, 160019 Shymkent, Kazakhstan; 14 Department of Neurology and Neurosurgery, National Institute of Health, Yerevan, Armenia; 15 Department of Physiology, Faculty of Medicine, Universiti Malaya, 50603 Kuala Lumpur, Malaysia; 16 The Global Parkinson’s Genetics Program (GP2), Bethesda, USA; 17 Department of Medicine, Faculty of Medicine, Universiti Kebangsaan Malaysia, Selangor, Malaysia; 18 Department of Neurology, National Taiwan University Hospital Taipei, Taipei, Taiwan; 19 Department of Neurology, Seoul National University Hospital, College of Medicine, Seoul National University, Seoul, Republic of Korea; 20 Department of Neurology, Seoul Metropolitan Government - Seoul National University Boramae Medical Center, Seoul National University College of Medicine, Seoul, Korea; 21 Department of Neurology, Asan Medical Center, University of Ulsan College of Medicine, Seoul, South Korea; 22 Duke-National University of Singapore Medical School, Singapore, Singapore; Department of Neurology, National Neuroscience Institute, Singapore General Hospital, Singapore, Singapore; 23 Preventive Neurology Unit, Wolfson Institute of Population Health, Queen Mary University of London, London, UK; 24 Department of Medicine, College of Medicine, University of Lagos, Lagos, Nigeria; 25 Sanatorio de la Trinidad Mitre-INEBA, Buenos Aires, Argentina; 26 Hospital JM Ramos Mejia, Buenos Aires, Argentina; 27 Somnus Neurology Clinic, Yerevan, Armenia; 28 Neuroscience Research Australia, Sydney, NSW Australia; 29 ANZAC Research Institute, Concord, NSW Australia; 30 Garvan Institute of Medical Research and Concord Repatriation General Hospital, Darlinghurst, NSW Australia; 31 Concord Hospital, Concord, NSW Australia; 32 QIMR Berghofer Medical Research Institute, Herston, QLD Australia; 33 Murdoch University, Perth, WA Australia; 34 Medical University of Vienna, Vienna, Austria; 35 Universidade Federal do Rio Grande do Sul / Hospital de Clínicas de Porto Alegre, Porto Alegre, Brazil; 36 University of São Paulo, São Paulo, Brazil; 37 Universidade Federal de Minas Gerais, Belo Horizonte, Brazil; 38 McGill University, Montreal, QC Canada; 39 Universidad de Chile, Santiago, Chile; 40 Fundación Diagnosis, Santiago, Chile; 41 Faculty of Medicine, Universidad de Chile, Santiago, Chile; 42 CETRAM, Santiago, Chile; 43 Central South University, Changsha, China; 44 West China Hospital, Sichuan University, Chengdu, China; 45 Xiangya Hospital, Central South University, Changsha, China; 46 Capital Medical University, Beijing, China; 47 Zhejiang University, Hangzhou, China; 48 Universidad Nacional de Colombia, Bogotá, Colombia; 49 Fundación Valle del Lili, Santiago de Cali, Colombia; 50 University of Antioquia, Medellín, Colombia; 51 University of Costa Rica, San José, Costa Rica; 52 The American University in Cairo, Cairo, Egypt; 53 Beni-Suef University, Beni Suef, Egypt; 54 Addis Ababa University, Addis Ababa, Ethiopia; 55 Paris Brain Institute, Paris, France; 56 Sorbonne Université, Paris, France; 57 University of Lübeck, Lübeck, Germany; 58 Deutsches Zentrum für Neurodegenerative Erkrankungen (DZNE), Göttingen, Germany; 59 University Medical Center Göttingen, Göttingen, Germany; 60 University Hospital LMU Munich, Munich, Germany; 61 Hannover Medical School, Hannover, Germany; 62 University Medical Center Schleswig-Holstein, Lübeck, Germany; 63 University of Tübingen, Tübingen, Germany; 64 German Center for Neurodegenerative Diseases (DZNE), Tübingen, Germany; 65 University of Mainz, Mainz, Germany; 66 University of Ghana Medical School, Accra, Ghana; 67 University of Thessaly, Larissa, Greece; 68 Aristotle University of Thessaloniki, Thessaloniki, Greece; 69 Ionian University, Corfu, Greece; 70 Biomedical Research Foundation of the Academy of Athens, Athens, Greece; 71 Diagnostic and Therapeutic Centre HYGEIA Hospital, Marousi, Greece; 72 Hospital San Felipe, Tegucigalpa, Honduras; 73 Queen Elizabeth Hospital, Kowloon, Hong Kong; 74 The Hong Kong University of Science and Technology, Kowloon, Hong Kong; 75 Aster Medcity, Kochi, India; 76 Sree Chitra Tirunal Institute for Medical Sciences and Technology, Thiruvananthapuram, India; 77 National Institute of Mental Health & Neurosciences (NIMHANS), Bengaluru, India; 78 Manipal Hospital, Delhi, India; 79 All India Institute of Medical Sciences, Delhi, India; 80 Nizam’s Institute of Medical Sciences, Hyderabad, India; 81 Shahid Beheshti University of Medical Sciences, Tehran, Iran; 82 Magna Græcia University of Catanzaro, Catanzaro, Italy; 83 University of Pavia, Pavia, Italy; 84 University of Perugia, Perugia, Italy; 85 University of Rome Tor Vergata, Rome, Italy; 86 Jikei University School of Medicine, Tokyo, Japan; 87 Institute of Neurology and Neurorehabilitation, Almaty, Kazakhstan; 88 Kyrgyz State Medical Academy, Bishkek, Kyrgyzstan; 89 Luxembourg Centre for Systems Biomedicine, University of Luxembourg, Belvaux, Luxembourg; 90 UKM Medical Molecular Biology Institute (UMBI), Kuala Lumpur, Malaysia; 91 Universiti Kebangsaan Malaysia Medical Centre, Kuala Lumpur, Malaysia; 92 Neuroscience Unit, Clinical Pharmacology Dept, Menoufia Medical School, Shebeen El-Kom, Egypt; 93 Instituto Nacional de Neurología y Neurocirugía, Mexico City, Mexico; 94 Universidad Nacional Autónoma de México, Mexico City, Mexico; 95 Mongolian National University of Medical Sciences, Ulaanbaatar, Mongolia; 96 Tribhuvan University, Kirtipur, Nepal; 97 University of Otago, Christchurch, New Zealand; 98 College of Medicine, University of Lagos, Lagos, Nigeria; 99 Norwegian University of Science and Technology, Trondheim, Norway; 100 Oslo University Hospital, Oslo, Norway; 101 University of Science and Technology Bannu, Khyber Pakhtunkhwa, Pakistan; 102 Instituto Nacional de Ciencias Neurológicas, Lima, Peru; 103 Metropolitan Medical Center, Manila, Philippines; 104 University of Puerto Rico, San Juan, Puerto Rico; 105 Research Center of Neurology, Moscow, Russia; 106 King Faisal Specialist Hospital and Research Center, Riyadh, Saudi Arabia; 107 King Abdullah International Medical Research Center, Jeddah, Saudi Arabia; 108 University of KwaZulu-Natal, Durban, South Africa; 109 University of Stellenbosch, Stellenbosch, South Africa; 110 Seoul National University Hospital, Seoul, South Korea; 111 Yongin Severance Hospital, Seoul, South Korea; 112 Hospital Universitario de Burgos, Burgos, Spain; 113 University Hospital Mutua Terrassa, Barcelona, Spain; 114 Institut de Recerca Sant Joan de Déu, Barcelona, Spain; 115 Research Institute Germans Trias i Pujol, Badalona, Spain; 116 University Hospital Germans Trias i Pujol, Badalona, Spain; 117 Faculty of Medicine, University of Khartoum, Khartoum, Sudan; 118 Inselspital, Bern University Hospital, University of Bern, Bern, Switzerland; 119 National Taiwan University Hospital, Taipei City, Taiwan; 120 Chang Gung Memorial Hospital, Taoyuan City, Taiwan; 121 National Taiwan University, Taipei City, Taiwan; 122 Chang Gung University, Taoyuan City, Taiwan; 123 National Institute Mongi Ben Hamida of Neurology, Tunis, Tunisia; 124 Mongi Ben Hmida National Institute of Neurology, Tunis, Tunisia; 125 Koç University, Istanbul, Turkey; 126 Şişli Etfal Training and Research Hospital, Istanbul, Turkey; 127 University College London, London, UK; 128 University of Plymouth, Plymouth, UK; 129 Parkinson’s UK, London, UK; 130 University of Glasgow, Glasgow, UK; 131 Queen Mary University of London, London, UK; 132 Cardiff University, Cardiff, UK; 133 Royal Veterinary College, University of London, London, UK; 134 University of Bristol, Bristol, UK; 135 Cure Parkinson’s Trust, London, UK; 136 University of Cincinnati, Cincinnati, OH USA; 137 The Michael J. Fox Foundation for Parkinson’s Research, New York, NY USA; 138 Augusta University / University of Georgia Medical Partnership, Athens, GA USA; 139 Mid-Atlantic Permanente Medical Group, Rockville, MD USA; 140 Washington University in St. Louis, St. Louis, MO USA; 141 National Institutes of Health, Bethesda, MD USA; 142 Indiana University, Bloomington, IN USA; 143 Rush University Medical Center, Chicago, IL USA; 144 Kaiser Permanente, Oakland, CA USA; 145 Aligning Science Across Parkinson’s, Washington, DC USA; 146 Banner Sun Health Research Institute, Sun City, AZ USA; 147 Michigan State University, East Lansing, MI USA; 148 Northwestern University, Chicago, IL USA; 149 Baylor College of Medicine, Houston, TX USA; 150 Baylor College of Medicine/Texas Children’s Hospital, Houston, TX USA; 151 University of Miami Miller School of Medicine, Miami, FL USA; 152 Beth Israel Deaconess Medical Center, Boston, MA USA; 153 NorthShore University HealthSystem, Evanston, IL USA; 154 Institute for Neurodegenerative Disorders, New Haven, CT USA; 155 University of Pittsburgh, Pittsburgh, PA USA; 156 University of Alabama at Birmingham, Birmingham, AL USA; 157 University of Maryland School of Medicine, Baltimore, MD USA; 158 University of Michigan, Ann Arbor, MI USA; 159 Columbia University Irving Medical Center, New York, NY USA; 160 James J. Peters VA Medical Center, Bronx, NY USA; 161 University of Chicago, Chicago, IL USA; 162 Indiana University School of Medicine, Indianapolis, IN USA; 163 Hue University, Huế, Vietnam; 164 University of Zambia, Lusaka, Zambia

**Keywords:** Diseases, Genetics, Neurology, Neuroscience

## Abstract

Common and rare variants in *LRRK2* influence Parkinson’s disease (PD) risk across diverse populations, and in this study, the rare p.A419V variant was investigated across multiple ancestry cohorts comprising over 200,000 PD cases and controls. In cases of East Asian (EAS) ancestry, p.A419V was significantly associated with increased risk of PD (OR = 2.9; 95% CI: 1.66–5.10; *p* = 0.0002), and was not in linkage disequilibrium with other *LRRK2* coding variants. The variant was significantly associated with a lower age at PD onset in the study cohort, while a meta-analysis of the EAS cases indicated a similar, albeit non-significant trend. LRRK2 protein modelling prediction indicated that binding sites for RAB8A, RAB29 and RAB32 were in close proximity to the p.A419V variant within the ARM domain. Together, these findings confirm the p.A419V as a significant PD risk factor in EAS populations, as well as highlight disease-relevant variants in the ARM domain and the link with LRRK2-RAB signaling.

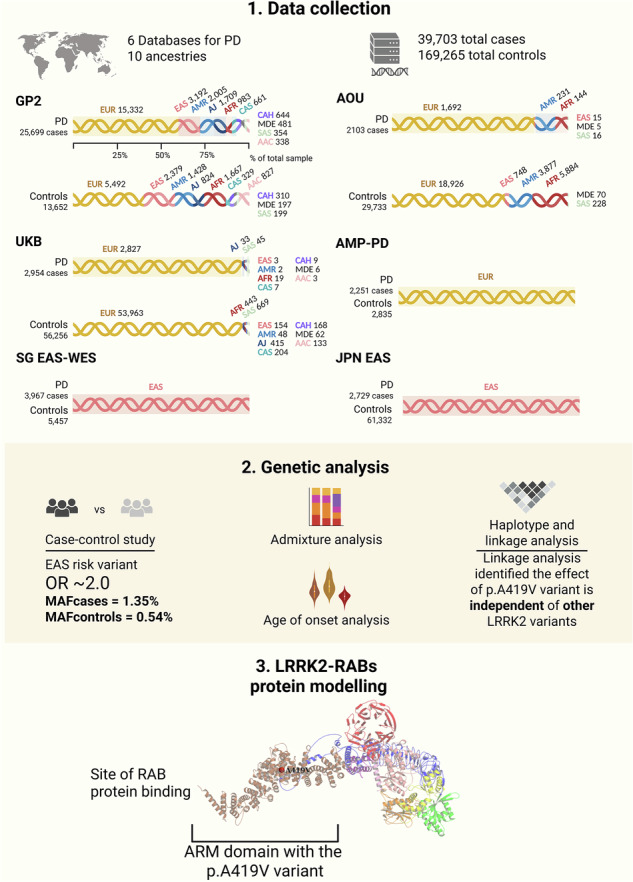

## Introduction

Pathogenic variants in the *LRRK2* gene, including p.R1067Q, p.N1437H, p.R1441G/C/H, p.Y1699C, p.G2019S, and p.I2020T, are known to cause Mendelian forms of Parkinson’s disease (PD), and several have also been identified as risk variants through genome-wide association studies (GWAS) and case-control analyses^1–4^. *LRRK2* ‘Asian risk variants’, p.R1628P and p.G2385R, have been identified as key risk factors for sporadic PD in various Asian populations^5,6^.

Another *LRRK2* variant, p.A419V (GRCh38, chr12:40252984:C > T, rs34594498, NM_198578.4:c.1256 C > T), was first studied as a potential PD-associated variant in East Asians (EAS) from Taiwan^7^, where no significant association was found in a cohort of 608 cases and 373 controls. In a subsequent, larger combined EAS cohort from Japan, Korea, and Taiwan (1376 cases; 962 controls), p.A419V was reported as a significant risk variant (odds ratio [OR] = 2.7; 95% confidence interval [CI] = 1.35–3.83; *p* = 0.0045)^1^. Several studies in EAS populations attempting to replicate this finding reported varied and inconsistent results^8–12^. More recently, this variant reached suggestive genome-wide significance in a mainland China PD GWAS^13^ and in EAS cohorts from 23andMe in a multi-ancestry meta-analysis^14^.

The p.A419V variant was not reported as significant in a case-control study from Kazakhstan in Central Asia^15^ (minor allele frequency, MAF = 3.7%, *n* = 292 cases, 199 controls; OR = 1.5, *p* = 0.4). In a South Asian cohort^16^, the p.A419V variant is rare with a MAF = 0.4% (*n* = 4806 cases, 6364 controls). The variant is absent in studies on Vietnamese^17^ (*n* = 83 early-onset PD (EOPD) cases), and there is no available information from recent studies in Japan^18^ (*n* = 221 cases), Thailand^19^ (*n* = 47 EOPD cases), or Korea^20^ (GWAS *n* = 1050 cases, 5000 controls). This variant is absent/rare in European (EUR)^14,21^ (*n* = 37,688 cases, 18,618 proxy-cases, 1.4 million controls; *n* = 49,049 cases, 18,785 proxy cases and 2,458,063 controls), African^22^ (GWAS *n* = 1488 cases; 196,430 controls), Latin American^23,24^ (1734 cases and 1097 controls; *n* = 807 cases and 690 controls), and Egyptian^25^ PD cohorts. These differences in *LRRK2* p.A419V detection may be due to the higher allele frequency of p.A419V in EAS (MAF = 0.01028) compared to MAF < 0.001 in European, South Asian, and Middle Eastern populations, and an even lower frequency in African populations (MAF < 0.0001), as reported in gnomAD v4.1.0.

LRRK2 activity assays indicate that the p.A419V variant increases LRRK2-mediated Rab10Thr73 phosphorylation by more than 1.5-fold compared to wild-type, and moderately elevates the formation of LRRK2 filaments in the absence of MLi-2 treatment, consistent with other pathogenic *LRRK2* variants^26^.

Despite evidence pointing to a potential deleterious effect of the *LRRK2* p.A419V variant, findings of variable significance across genetic association studies have limited its inclusion in further functional characterization and clinico-genetic correlation efforts, particularly in comparison to the well-established EAS *LRRK2* variants such as p.G2385R and p.R1628P^5^. The relevance of this variant in other PD populations has not been not fully studied. To address this gap while also increasing confidence for deep phenotyping and precision medicine applications, we leveraged large-scale genotyping data from the Global Parkinson’s Genetics Program (GP2), whole-genome sequencing (WGS) data from the Accelerating Medicines Partnership Parkinson’s Disease (AMP-PD) program^27,28^, the UK Biobank (UKB, www.ukbiobank.ac.uk), and the All of Us Research Program (AOU, https://allofus.nih.gov/), as well as whole-exome sequencing (WES) data from a Singapore (SG) EAS dataset^29^ and Juntendo University Hospital PD cohort. In addition, there is inconclusive evidence of the influence of Asian *LRRK2* risk variants on age at PD onset (AAO)^30,31^; therefore, this study sought to address this further for the p.A419V variant.

## Results

### Overall data review

Demographics for each cohort separated by ancestry, including age at sample collection, age at onset, sex, and number of cases/controls, are presented in Supplementary Table 1. Overall, the proportion of males and females is comparable across ancestries and cohorts. As expected for a well-designed case–control study, the mean age of controls is generally older than or comparable to that of PD cases. The only exception is the CAH cohort in GP2, where the mean control age is below 50 years, which may dilute the statistical power in analyses involving age-related traits. The *LRRK2* p.A419V variant was in HWE across all ancestry groups in all the cohorts (Supplementary Table 3).

In the GP2 cohort, the *LRRK2* p.A419V variant was found to be rare (MAF < 1%) across all populations (Table 1), with the highest frequency observed in the EAS (MAF = 1.3% cases, 0.36% controls), followed by CAS (MAF = 0.98% cases, 0.6% controls), CAH (MAF = 1.1% cases, none in controls), EUR (MAF = 0.12% cases, 0.02% controls), AJ (MAF = 0.03% cases, none in controls). In contrast, no carriers were observed in the AAC, AFR, AMR, MDE, and SAS individuals. None of the cases were carriers in the EUR AMP-PD cohort, while the MAF in controls was 0.02%. In the UKB cohort, no carriers were identified in AAC, AFR, AMR, CAH, CAS, FIN, or MDE ancestry groups, while the MAF in EAS controls was 0.65% and no carriers were identified in the six EAS cases. The variant was present in UKB EUR individuals with MAF = 0.02% (cases) and MAF = 0.01% (controls). The MAF in the UKB SAS was 1.1% cases and 0.08% controls. In the AOU cohort, the highest number of carriers were of EAS (MAF = 6.60% cases, 0.40% controls), followed by EUR (MAF = 0.03% cases, 0.03% controls) and AMR (MAF = 0.01% controls, none in cases) ancestries.

**Table 1:** Frequency of the *LRRK2* p.A419V variant across ancestries

Dataset	Ancestry	PD, n (%) breakdown in cohort	Control, n (%) breakdown in cohort	N Total Carriers	AF Total (AC)	N PD Carriers	AF PD cases (AC)	N Control Carriers	AF controls (AC)	AF gnomAD
**GP2 25,699 cases; 13,652 controls**	**AAC**	338 (29.01)	827 (70.99)	0	0 (0/2330)	0	0 (0/676)	0	0 (0/1654)	NA
	**AFR**	983 (37.09)	1667 (62.91)	0	0 (0/5300)	0	0 (0/1966)	0	0 (0/3334)	0.00001334
	**AJ**	1709 (67.47)	824 (32.53)	1	0.00020 (1/5066)	1	0.00029 (1/3418)	0	0 (0/1648)	0
	**AMR**	2005 (58.4)	1428 (41.6)	0	0 (0/6866)	0	0 (0/4010)	0	0 (0/2856)	0.00006669
	**CAH**	644 (67.51)	310 (32.49)	14	0.0073 (14/1908)	14	0.011 (14/1288)	0	0 (0/620)	NA
	**CAS**	661 (66.77)	329 (33.23)	17	0.0086 (17/1980)	13	0.0098 (13/1322)	4	0.006 (4/658)	NA
	**EAS**	3192 (57.3)	2379 (42.7)	97	0.0089 (99/11,142)	81*	0.013 (82/6384)	16*	0.0036 (17/4758)	0.01028
	**EUR**	15,332 (73.63)	5492 (26.37)	37	0.00094 (39/41,648)	35 ^#	0.0012 (37/30,664)	2	0.00018 (2/10,984)	0.0001493
	**MDE**	481 (70.94)	197 (29.06)	0	0 (0/1356)	0	0 (0/962)	0	0 (0/394)	0.0008267
	**SAS**	354 (64.01)	199 (35.99)	0	0 (0/1106)	0	0 (0/708)	0	0 (0/398)	0.0001977
**AMP-PD 2835 cases; 2.251 controls**	**EUR**	2251 (44.26)	2835 (55.74)	1	0.000098 (1/10,172)	0	0 (0/4502)	1	0.00018 (1/5670)	0.0001493
**UKB 2954 cases; 56,256 controls**	**AAC**	3 (2.21)	133 (97.79)	0	0 (0/272)	0	0 (0/6)	0	0 (0/266)	NA
	**AFR**	19 (4.11)	443 (95.89)	0	0 (0/924)	0	0 (0/38)	0	0 (0/886)	0.00001334
	**AJ**	33 (7.42)	412 (92.58)	0	0 (0/890)	0	0 (0/66)	0	0 (0/824)	0
	**AMR**	2 (4)	48 (96)	0	0 (0/100)	0	0 (0/4)	0	0 (0/96)	0.00006669
	**CAH**	9 (5.08)	168 (94.92)	0	0 (0/354)	0	0 (0/18)	0	0 (0/336)	NA
	**CAS**	7 (3.32)	204 (96.68)	0	0 (0/422)	0	0 (0/14)	0	0 (0/408)	NA
	**EAS**	3 (1.91)	154 (98.09)	2	0.0064 (2/314)	0	0 (0/6)	2	0.0065 (2/308)	0.01028
	**EUR**	2827 (4.98)	53963 (95.02)	10	0.000088 (10/113,580)	1	0.00018 (1/5654)	9	0.000083 (9/107,926)	0.0001493
	**MDE**	6 (8.82)	62 (91.18)	0	0 (0/136)	0	0 (0/12)	0	0 (0/124)	0.0008267
	**SAS**	45 (6.3)	669 (93.7)	2	0.0014 (2/1428)	1	0.011 (1/90)	1	0.00075 (1/1338)	0.0001977
**AOU 2103 cases; 29,733 controls**	**AFR**	144 (6.8)	5884 (19.8)	0	0 (0/12,052)	0	0 (0/288)	0	0 (0/11,764)	0.00001334
	**AMR**	231 (11)	3877 (13)	1	1.22E-04 (1/8216)	0	0 (0/462)	1	0.0001 (1/7754)	0.00006669
	**EAS**	15 (0.7)	748 (2.5)	8	0.005 (8/1526)	2	0.066 (2/30)	6	0.004 (6/1496)	0.01028
	**EUR**	1692 (80.4)	18,926 (63.7)	13	3.15E-04 (13/41,236)	1	2.96E-04 (1/3384)	12	3.17E-04 (12/37,852)	0.0001493
	**MDE**	5 (0.2)	70 (0.2)	0	0 (0/150)	0	0 (0/10)	0	0 (0/140)	0.0008267
	**SAS**	16 (0.8)	228 (0.8)	0	0 (0/488)	0	0 (0/32)	0	0 (0/456)	0.0001977
**Singapore EAS-WES 3967 cases; 5457 controls**	**EAS**	3967 (42.09)	5457 (57.91)	177	0.0058 (180/18,848)	107”	0.0139 (110/7934)	70	0.0064 (70/10,914)	0.01028
**JPN 2729 cases; 61,332 controls**	**EAS**	2729 (4.26)	61,332 (95.74)	2086	0.0165 (2,107/128,112)	167**‘**	0.0321 (175/5458)	1915**“**	0.0158 (1,932/122,664)	0.01028

*Include 1 homozygous carrier.

^Include 2 homozygous carriers.

“Include 3 homozygous carriers.

'Include 8 homozygous carriers.

"Include 17 homozygous carriers.

#22 out of 35 of these patients were submitted from Central Asia.

*AC* Allele Counts, *AF* Allele Frequency, *AF gnomAD* Population specific allele frequency in gnomAD 4.1, *AAC* African American or Caribbean, *AFR* Sub-Saharan African, *AJ* Ashkenazi Jewish, *AMR* Admixed American, *CAH* Complex Admixture History, *CAS* Central Asian, *EAS* East Asian populations, *EUR* European, *MDE* Middle Eastern, *SAS* South Asian.

Family history was not more commonly reported in *LRRK2* p.A419V carriers (17.7%) compared to PD non-carriers across all ancestries, where it was reported in 25.2% of individuals (Table 1). A higher frequency of female *LRRK2* p.A419V carriers was observed in the GP2 CAS and EUR groups (CAS: *p* = 0.008, 92.3% in carriers vs. 53.7% in non-carriers; EUR: *p* = 0.041, 54.6% in carriers vs. 37.7% in non-carriers) (Table 1).

### Risk association analysis

Power calculations indicated that only the EAS and EUR cohorts in GP2 had an 80% power to detect an association with an OR > 2.0 and a *p* value < 0.05 (Supplementary Table 4), while the AMP-PD, UKB, and AOU cohorts were underpowered for association analysis of the *LRRK2* p.A419V variant.

Logistic regression analyses conducted in the GP2 EAS and EUR ancestry groups indicated that a significantly higher frequency of the *LRRK2* p.A419V variant was observed in cases compared to controls in both the EAS (MAF cases = 1.15% vs. MAF controls = 0.57%; OR = 2.908; 95% CI = 1.659–5.098, *p* = 0.0002) and EUR (MAF cases = 0.06% vs. MAF controls = 0.02%; OR = 5.754; 95% CI = 1.399–23.66, *p* = 0.015) groups (Supplementary Table 5). However, the association in the EUR group was further investigated, as described below in ‘Admixture analysis of the GP2 EUR cohort’. The association in the GP2 EAS group was tested in two independent EAS cohorts. In the Singapore-EAS exome dataset^29^, the *LRRK2* p.A419V variant was present in 107 of 3967 cases (1.4%) and 70 of 5457 controls (0.6%), with a significant risk association (OR = 1.51, 95% CI = 1.103–2.068, *p* = 0.012, Supplementary Table 6). In the Juntendo EAS-Japanese replication cohort, the p.A419V variant was significantly associated as a risk factor in Japanese PD patients (MAF = 3.20%) than in the controls (MAF = 1.58%); (OR = 2.06; 95% CI: 1.76–2.42; *p* = 1.301 × 10⁻16, Supplementary Table 6).

In the GP2 cohort, there was minimal LD (r² < 0.01) between p.A419V and other *LRRK2* coding variants across all ancestries studied (Supplementary Table 7), suggesting that the observed risk association is unlikely to be confounded by nearby coding variation. In addition, the *LRRK2* p.A419V variant is not in LD with any lead SNPs identified in EAS PD GWAS^4,14,32,33^. The LD block constructed around p.A419V consisted of 8 SNPs (rs10506148, rs28365214, rs10878249, p.A211V, rs732374, p.V366M, p.L378F, rs1491938). Six rare haplotypes (MAF < 0.01) were identified in the GP2 EAS cohort, with one (Haplotype 9) observed in 1.2% PD cases and 0.34% controls (Supplementary Table 8), but further analysis lacked sufficient power to assess association. The haplotype analysis was not performed in the Singapore EAS-WES dataset, as its exome sequencing data would not have fully contained sufficient variants to perform the *LRRK2* haplotyping effectively. Additionally, the UKB and AOU cohorts had relatively small EAS sample sizes, limiting their utility for this analysis. No haplotype containing *LRRK2* p.A419V was identified in the other GP2 ancestries due to the rarity of the variant.

### Admixture analysis of the GP2 EUR cohort

As the comparatively higher allele frequency and risk association of the *LRRK2* p.A419V in European individuals was unique to the GP2 EUR cohort - unlike the other EUR cohorts from AMP-PD, UKB, and AOU—carriers in the GP2 group were examined further. Based on ADMIXTURE analysis, the GP2 EUR *LRRK2* p.A419V carriers were found to be highly admixed, with 29/35 carriers having relatively lower EUR ancestry (median: 13.86%, range: 4.68–70.8%, Supplementary Table 9a) compared to EUR non-carriers (median: 65.75%, range: 4.09–82.30%, Supplementary Table 9b). Notably, 22/35 carriers in the EUR ancestry group were Kazakhstani Central Asian patients. Therefore, these carriers may not be fully representative of the EUR ancestry group. In line with this, linear regression against percentage of genomic admixture in this group indicated that individuals who have less EUR ancestry are more likely to be *LRRK2* p.A419V carriers (β = -0.23, SE = 1.78; *p* = 3.01e–19). In contrast, all the EAS p.A419V carriers demonstrated high EAS ancestry (median: 91.68%, range: 71.9–94.1%, Supplementary Table 9c) similar to EAS non-carriers (median: 91.83%, range: 44.24–94.91%, Supplementary Table 9d), lending greater confidence to the observed associations within this population.

### Age at onset association analysis in EAS cohorts

**Fig. 1:**
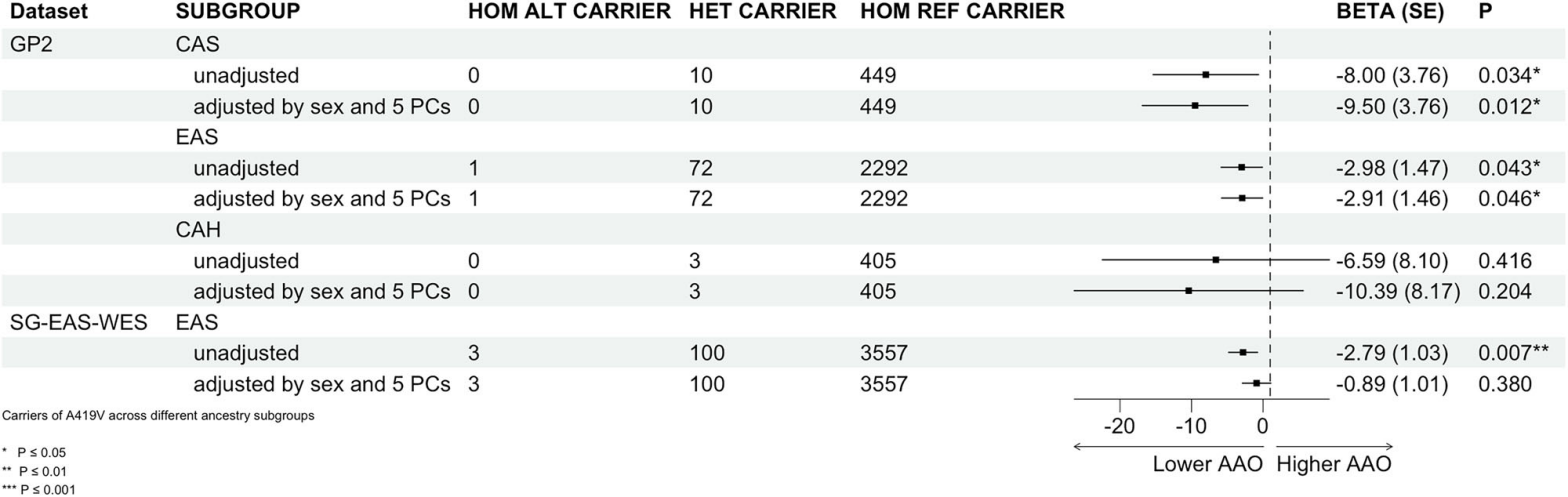
*LRRK2* p.A419V and age at onset. Association of *LRRK2* p.A419V with age at onset were conducted using generalized linear models (GLM) with linear regression under an additive model.

Analysis of *LRRK2* p.A419V carrier status and AAO in the GP2 EAS PD group (*n* = 73 carriers, AAO 50.3 ± 12.6 years vs. *n* = 2292 non-carriers, AAO 53.4 ± 12.7 years) (Table 2), revealed an association with an earlier onset of PD by approximately 3 years (β = –3.02 years; SE = 1.49; *p* = 0.043) after adjusting for sex and 5 PCs (Fig. 1). Six *LRRK2* p.A419V carriers also carried a concomitant p.G2385R variant (mean AAO: 51.3 ± 13 years), and one patient with young onset, unknown monogenic PD status (AAO: 35 years), was found to have a concomitant p.R1628P variant (Supplementary Table 10). In the replication Singapore EAS-WES cohort, which included 103 *LRRK2* p.A419V PD carriers and 3557 non-carriers, linear regression adjusted for sex showed a similarly significant reduction in disease onset by approximately two and a half years (β = –2.79 years, SE = 1.03; *p* = 0.007). However, after additional adjustment for the first three PCs, the association was no longer statistically significant (β = –0.89 years; SE = 1.01; *p* = 0.380), despite the directionality of effect remaining consistent, with carriers showing a lower AAO (Fig. 1). A fixed-effects meta-analysis combining the discovery and replication cohorts demonstrated a reduced but borderline-significant association (β = –1.55 years, SE = 0.83, *p* = 0.063; Supplementary Fig. 3), supporting a modest effect of the variant on age at onset.

**Table 2:** Age at PD onset between *LRRK2* p.A419V PD carriers and non-carriers in the GP2 cohort

	Total			AJ			CAS			CAH			EUR			EAS		
	p.A419V carrier	non-carrier	*p* value	p.A419V carrier	non-carrier	*p* value	p.A419V carrier	non-carrier	*p* value	p.A419V carrier	non-carrier	*p* value	p.A419V carrier	non-carrier	*p* value	p.A419V carrier	non-carrier	*p* value
**AAO, N**	100	15,189	2.526e–09***	1	1338		10	449	0.035*	3	405	0.520	13	10,705	0.357	73	2292	0.028*
**mean** ± **SD**	50.29 ± 12.70	57.95 ± 12.40		45	62.37 ± 11.59		44.8 ± 11.85	52.8 ± 11.75		47.33 ± 17.67	53.93 ± 13.95		55.46 ± 12.93	58.74 ± 12.03		50.32 ± 12.56	53.41 ± 12.65	
**median [IQR]**	49 [20]	59 [18]		45	64 [16]		48.5 [16]	54 [16]		56 [16]	55 [19]		55 [16]	60 [16 .4]		49 [20]	52 [18]	
**FHX, n/N**	14/79	4251/16,848	0.142	0/0	484/1572	NA	1/11	58/505	1	2/4	100/504	0.181	3/11	2953/11,610	0.356	8/53	656/2657	0.291
**(%)**	17.72	25.23		0	30.79		9.09	11.49		50	19.84		27.27	25.43		15.09	24.69	
**Male, n/N**	57/141	11,922/19,675	0.000***	1/1	1125/1705	1	1/13	300/648	0.008**	4/14	365/630	0.052	15/33	9528/15,288	0.041*	37/81	1729/3109	0.087
**(%)**	40.43	60.59		100	65.98		7.69	46.3		28.57	57.94		45.45	62.32		45.68	55.61	

Mann–Whitney U test was used for comparing age at onset difference between carrier and non-carrier groups. Fisher’s test was used for family history and sex comparison between carrier and non-carrier groups.

*n* number of individuals with available information, *N* total number of individuals, *IQR* inter quartile range, *FHX* family history of PD, *SD* standard deviation, *AAO* age at onset, *AJ* Ashkenazi Jewish, *CAH* Complex Admixture History, *CAS* Central Asian, *EAS* East Asian, *EUR* European.

*P ≤ 0.05; ** *P* ≤ 0.01; *** *P* ≤ 0.001

The CAS ancestry in the GP2 cohort was observed to show a significant association with earlier AAO (β = –4.25 years; SE = 8.173; *p* = 0.011). However, given the small number of carriers in this group (*n* = 10), this should be interpreted cautiously. No significant association between *LRRK2* p.A419V and AAO was observed in the CAH group (only present in the GP2 cohort), before or after adjusting for covariates.

### Structural modeling of LRRK2 and its RAB binding interfaces

**Fig. 2:**
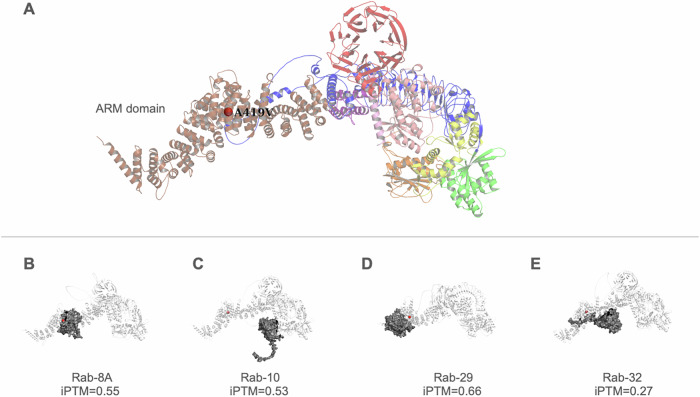
Mutation sites and predicted RAB binding of LRRK2. **A** Visualization of the predicted LRRK2 structure with different colored domains (armadillo repeat (ARM) domain in brown/tan, ankyrin repeat (ANK) domain in pink, leucine-rich repeat (LRR) domain in blue, ROC (Ras of complex proteins) GTPase domain in yellow, COR (C-terminal of ROC) domain in orange, kinase domain in green, and the WD40 domain in red). The alanine residue at position 419, substituted by valine in the p.A419V variant, is indicated by a red sphere within the ARM domain. Binding predictions of the different RAB proteins with LRRK2: **B** RAB8A, **C** RAB10, **D** RAB29, **E** RAB32.

Visualization of the predicted LRRK2 structure overlaid with known variants revealed that the p.A419V variant resides within the ARM domain (amino acid position 12–704)^34^ (Fig. 2A). Predicted structures of the LRRK2-RAB complexes demonstrated that the binding sites for RAB8A, RAB29, and RAB32 are spatially proximal to the p.A419V mutation site, consistent with experimental observations (Fig. 2B, D, E). Notably, the predicted binding site for RAB10 (Fig. 2C) is not within the ARM domain, despite experimental evidence suggesting similar binding motifs as with the other Rab proteins^35,36^.

## Discussion

We investigated the genetic association between the *LRRK2* p.A419V variant, PD risk, and AAO, leveraging data across several cohorts including the multi-ancestry GP2, AMP-PD, UKB, AOU cohorts, as well as a Singapore-EAS exome dataset and a Japanese-EAS dataset, totaling 40,287 PD cases and 168,681 controls. This study resolves inconsistencies around previous reports by providing robust evidence that the *LRRK2* p.A419V is significantly associated with PD in the EAS population, with an approximate OR of 2.0. This is comparable with other *LRRK2* EAS risk variants, p.G2385R (OR ~ 2.3)^37^ and p.R1628P (OR ~ 1.8)^38^.

The variant is rare in EUR (MAF = 0.03%) and even rarer or absent in AAC, AFR, AJ, AMR, FIN, and MDE populations. Compared to the other *LRRK2* risk variants p.R1628P and p.G2385R (MAFs between 5-10% in cases and 2–5% in controls)^39^, in the overall EAS cohorts in this study, the p.A419V variant is much less common (MAF_Total_ = 0.91%, MAF_cases_ = 1.35% and MAF_controls_ = 0.54%). Interestingly, this variant was also seen in other Asian populations, such as CAS (MAF_Total_ = 0.71%, MAF_cases_ = 0.97% and MAF_controls_ = 0.38%) and SAS (MAF_Total_ = 0.07%, MAF_cases_ = 0.12% and MAF_controls_ = 0.05%), compared to non-Asian populations, suggesting this variant may be more relevant to individuals of ‘East-Central-South’ Asian ancestries. The MAF seen in the SAS population in this study (0.07%) compares to 0.4% in a SAS GWAS by Kishore et al. ^16^ (*n* = 11,170), with gnomAD reporting an MAF 0.02%. Thus far, the p.A419V variant has not been investigated as a risk variant in SAS populations. In CAS populations, a previous study found no significant association between the p.A419V variant and PD^15^, and this has also been observed in an unpublished extended sample set of 655 cases (MAF = 1.38%) and 528 controls (MAF = 0.85%) (personal communication, RK).

Only one *LRRK2* p.A419V haplotype (Haplotype 9) was seen in both cases and controls, but association analysis for this haplotype is not possible due to its low frequency in both cases and controls. No disease-specific haplotype enrichment was observed. Haplotype analysis was not performed in the Singapore EAS-WES cohort due to limited variants from exome data.

Regarding the heritability of AAO concerning *LRRK2* risk variants, a directionality towards an earlier AAO has been reported - specifically, the combination of the p.G2385R-p.R1628P-p.S1647T variants was found responsible for lowering the AAO by approximately 8 years^30^. Regarding the *LRRK2* p.A419V variant, a previous EAS study of 2,685 cases from mainland China did not find any association with an earlier AAO^31^. However, our study showed a modest effect on AAO in the GP2 EAS and EAS exome cohorts. We found *LRRK2* p.A419V carriers developed PD approximately 3 years earlier than non-carriers, although we note the lack of robust association. A limitation of interpreting this result is the analysis of a single variant in one gene while other studies have used a GWAS approach to address the genetic contribution to AAO for PD. In European PD GWAS, variants in the *TMEM175* and *SNCA* genes^40^ and *BST1*^41^ were found to be associated with an earlier AAO, while a GWAS meta-analysis in EAS PD populations highlighted a SNP (rs9783733) in the novel *NDN*; *PWRN4* locus, delays AAO by 2.43 years, most significantly in male patients, as well as a suggestive signal in *SNCA* (rs3775458), which lowers AAO by 1.36 years^42^. More recently, SNPs in LD with *ALCAM* were associated with an earlier AAO by 3.47 years in a Korean AAO-GWAS^43^. Additionally, polygenic risk scores (PRS) across multiple studies have shown a strong inverse correlation with AAO^42–46^, thus the contribution of the *LRRK2* p.A419V variant on AAO will need to be evaluated more deeply in the context of other genome-wide genetic modifiers.

While an association with *LRRK2* p.A419V was initially found in the GP2 EUR group, closer inspection of these carriers revealed that 22/35 of these patients were of Central Asian origin. As this variant is very rare in Europeans and in Central Europeans^47^ and its association with PD has not been reported in individuals of EUR ancestry despite multiple GWAS and *LRRK2* studies^4,14,21,48^, we postulate that the association in our study may be an artifact due to inheritance of the *LRRK2* p.A419V variant from the EAS/CAS chromosomal regions in these individuals. Resolution of admixture in the EUR-CAS individuals could be performed through local ancestry analysis, however this was not currently possible as the number of control CAS genomes was insufficient. The issue of limited control individuals is not uncommon in underrepresented populations^49^ and presently poses a challenge in interpreting the contribution of rare variants such as p.A419V. Large-scale initiatives such as GP2, which is sequencing both PD cases and ancestry-matched controls, and existing or emerging national genome projects of several underrepresented populations contribute to bridging this gap. These include Kazakhstan (Central Asia)^50–52^, China (East Asia)^53^, Singapore (Southeast Asia)^54^, and the Americas^6,55^. Future efforts will play a crucial role in providing a more accurate interpretation of disease-relevant genomic variants.

The alanine residue at position 419 in LRRK2 is highly conserved (ConSurf score 9/9), suggesting that mutations at this site may significantly alter protein function. The p.A419V variant lies within the LRRK2 armadillo repeat domain (ARM), which mediates the interaction between LRRK2 and multiple Rab GTPases^36,56,57^. Thus, facilitating recruitment of LRRK2 to intracellular vesicle trafficking pathways, including the trans-Golgi network and endolysosomal system^58–61^. Recent studies indicate that the variant is located in a distinct region in the ARM domain, termed site #1 (encompassing amino acids 360–450), which is reported to be the precise binding site of RAB29, RAB8A, and RAB10^44,45^. Together, the RAB substrates anchor LRRK2 to membranes, enabling it to acquire a more active state through enhanced GTPase phosphorylation, in a proposed ‘feed-forward pathway’^35,62^. Cells carrying variants neighboring to p.A149V, p.L403A, and p.K439E interfere with optimal binding of RAB8A and RAB29 substrates^35^, and recently the p.[L119P;L488P] variants were also shown to affect the binding affinity of RAB8A^63^, and it could be postulated that the p.A419V variant may have similar effects. However, this has yet to be demonstrated and would be best addressed with in vitro and in vivo models.

Through LRRK2 protein modeling with Alphafold3, the predicted model with the available regions of the X-ray structure as published in Myasnikov et al., ^34^, yielded acceptable agreement (RMSD = 0.992) (Supplementary Fig. 4), underscoring the value of the predicted model for visualizing the ARM domain. The predicted interaction interfaces between LRRK2 and RAB proteins placed the p.A419V variant in close proximity to the binding regions for RAB8A, RAB29, and RAB32, which tallies with experimental data^35,36^. However, while these predictions offer valuable insights, they are inherently limited in defining the precise binding sites. Despite experimental evidence suggesting similar ARM domain binding motifs as seen with other RAB proteins^35,36^, the predicted binding outside the ARM domain for RAB10 may arise due to the AlphaFold3 model itself and/or the possibility that LRRK2 exists as a dimer during RAB protein interactions. Although these limitations exist, the predicted structures are useful for visualization and will guide future structural and functional investigations, particularly as current X-ray structures lack the ARM domain. This finding suggests if the p.A419V variant causes conformational changes to LRRK2, this may have an effect on binding affinity to RAB proteins through the ARM domain, and this may lead to disrupted LRRK2-RAB signaling. However, further experimental validation is needed to determine whether the p.A419V substitution has any effect on RAB interactions or LRRK2 kinase activity.

In conclusion, the *LRRK2* p.A419V variant is a rare but significant risk factor for PD in EAS individuals. These results highlight the need for the *LRRK2* p.A419V variant to be considered alongside the other *LRRK2* p.G2385R and p.R1628P risk variants in the context of Asian PD, and inclusion in comprehensive deep phenotyping efforts, as described in a recent publication on p.R1628P and p.G2385R on the utility of using genetic profiles for disease prognostication and management towards the aim for personalized precision medicine^5^. In addition, this study highlights that variants in the ARM domain, the site of several RAB substrate binding, may be a rare but relevant target for further clinical, biomarker, and therapeutic studies.

## Methods

### Cohorts under study

This study included six cohorts spanning multiple ancestries (Supplementary Table 1). Cohort 1 included genotyped data from GP2 release 9 (https://gp2.org/), comprising 25,699 unrelated PD patients and 13,652 controls from ten ancestry populations: European (EUR), East Asian (EAS), Admixed American/Latin American (AMR), Ashkenazi Jews (AJ), Central Asian (CAS), Complex Admixture History (CAH), Middle Eastern (MDE), South Asian (SAS), African American (AAC) and African (AFR). Cohort 2 was composed of data from AMP-PD WGS release 3 (https://amp-pd.org/), which included 2251 unrelated PD cases and 2835 controls of European descent. Cohort 3 comprised individuals from the UKB (2954 cases and 56,256 controls). Cohort 4 included data from the AOU Program (2103 cases, 29,733 controls). Cohort 5 was a Singapore EAS-WES replication cohort (3967 cases, 5457 controls) described further below. Cohort 6 was an EAS cohort of Japanese ancestry (2729 PD cases recruited from Juntendo University Hospital and 61,332 control individuals from the public database jMorp 61KJPN^64^, https://jmorp.megabank.tohoku.ac.jp/).

### Ethical considerations

This study was conducted in accordance with the ethical standards of the institutional and national research committees that has been reviewed and approved by Operations and Compliance Working Group (OCWG) of GP2. Additionally, sample providers have to share their consent documents which are also reviewed by the OCWG of GP2 to ensure that international sample and data sharing is allowed and that local data sharing restrictions are respected. Written informed consent is obtained at each individual site according to the local ethics protocol approved by the OCWG. Ethical approvals for the EAS replication cohorts are as such: Singapore: SingHealth Centralized Institutional Review Board (CIRB 2002/008/A and 2019/2334) and Nanyang Technological University Institutional Review Board (IRB-2016-08-011); Japan: Ethics committee of Juntendo University, Tokyo, Japan (M08-0477-M09). Ethics approval for the CAS cohort was obtained from the International Genetics Collaboration (IGC) CI: Prof H Houlden Sponsor EDGE ID: 146653 REC Ref: IRAS: 310045 Protocol V1.1 22/06/2022.

### Data quality control

Quality control (QC) of the GP2 data was performed using the GenoTools pipeline (https://github.com/GP2code/GenoTools)^65^. Briefly, samples were excluded if they had a genotyping rate below 95%, exhibited sex mismatches, or were duplicated (KINSHIP > 0.354), or displayed high heterozygosity ( | F| statistic >0.25). Variants were excluded if they had >5% missingness, significant deviations from Hardy-Weinberg Equilibrium (HWE *p* < 1e–4), or non-random missingness based on case-control status (*p* ≤ 1e–4). Ancestry estimation was performed using Genotools^65^, with the default reference panels from the 1000 Genomes Project, the Human Genome Diversity Project, and Ashkenazi Jewish datasets. Additionally, individuals with second-degree or closer relatedness (KINSHIP > 0.0884) were removed before analysis. The percentage of ancestry was then calculated using the supervised functionality of ADMIXTURE (v1.3.0; https://dalexander.github.io/admixture/binaries/admixture_linux-1.3.0.tar.gz)^66^ with the same reference panel mentioned before to estimate the ancestry proportions of the GP2 data accurately.

### Statistical analyses

Raw genotypes of the *LRRK2* p.A419V variant were extracted from the NeuroBooster Array v1.0 (NBA)^67^ (Supplementary Fig. 1) from GP2 data release 9 and from WGS data for AMP-PD version 3, the UKB, and the AOU.

*LRRK2* p.A419V PD carriers and non-carriers were compared for sex and family history using the two-tailed Fisher’s exact test. Association between the *LRRK2* p.A419V variant and PD risk was assessed by performing a logistic regression (glm) under an additive genetic model in PLINK 2.0^68^, here genotypes were encoded as 0 (homozygous major allele), 1 (heterozygous), and 2 (homozygous minor allele). Covariates, including sex and the appropriate number of principal components (PCs) were included in the model. The number of PCs was determined by identifying the elbow point of the scree plot (Supplementary Fig. 2) to account for population stratification (Supplementary Table 2)^69^. Additionally, allele and genotype frequencies, as well as HWE, were calculated using PLINK 2.0. Power calculation was performed using the GAS power calculator (https://csg.sph.umich.edu/abecasis/cats/gas_power_calculator/). The additive model was used with a significance level set at *p* = 0.05, PD general population prevalence of 0.5%^21^ at an OR of 2.27 according to the meta-analysis of this variant in the Asian population^1^ and an OR of 2.01 according to the largest Chinese GWAS study^13^. The association between p.A419V and AAO was assessed using a linear regression additive genetic model adjusted for sex and the appropriate number of PCs, as described above. Meta-analysis of AAO between GP2 EAS discovery cohort and Sg EAS-WES replication cohort was analysed using R meta package (v8.2-1; https://cran.r-project.org/web/packages/meta/index.html)^70^.

The Singapore EAS replication cohort consisted of published WES data^29^ from 3967 PD patients and 5457 ancestry- and geographically-matched controls from five regions across East Asia [Singapore (SG): 1955 cases, 3630 controls; Malaysia (MAL): 325 cases, 59 controls; Hong Kong (HK): 70 cases, 586 controls; South Korea (KR): 1417 cases, 1040 controls; Taiwan (TW): 200 cases, 142 controls]. A stratified Cochran-Mantel-Haenszel (CMH) test was used to evaluate the burden of *LRRK2* p.A419V across the exomes of participants from the replication cohort. Fisher’s exact test (two-tailed) was used to assess the burden of *LRRK2* p.A419V within each stratum. SG and MAL samples were considered as one stratum due to the similarity in genetic background.

Cohort 6 was a second EAS replication cohort of Japanese ancestry (2,729 PD cases recruited from Juntendo University Hospital with age at onset ≥21 and 61,000 control individuals from the public database jMorp 61KJPN^64^, https://jmorp.megabank.tohoku.ac.jp). Genotype comparisons in the Japanese population were conducted using the R software, including a two-tailed Fisher’s exact test for calculation of odds ratios (OR) for allele frequencies, and 95% confidence intervals (CI).

To characterize the linkage disequilibrium (LD) structure, pairwise r² values between *LRRK2* p.A419V and all *LRRK2* missense variants and other reported GWAS variants in the *LRRK2* locus^1–4,14,32,33^ were calculated using PLINK 1.9. Haplotype blocks in the GP2 EAS cohort were defined using the --block function in PLINK 1.9, with the minimum MAF threshold set to 0.0001 to allow the inclusion of *LRRK2* p.A419V across all ancestries. Haplotype frequency estimation and association analysis were performed using the haplo.stats R package (v1.9.7; https://cran.r-project.org/web/packages/haplo.stats/) under default settings.

### LRRK2 protein structure prediction

The three-dimensional structures of LRRK2, and complexes with RAB8A, RAB10, RAB29, RAB32 were predicted using AlphaFold3 (https://hpc.nih.gov/apps/alphafold3/)^71^ on the NIH Biowulf high-performance computing cluster (https://hpc.nih.gov). FASTA sequences for each protein (LRRK2:Q5S007, RAB8A:P61006, RAB10:P61026, RAB29:O14966, RAB32:Q13637) were retrieved from UniProt (https://www.uniprot.org/)^72^, and subsequently AlphaFold3 input JSON files were generated. Multiple sequence alignments and model inference were performed to generate predicted protein structures. To improve the robustness of the predictions, five independent runs were initiated using different random seeds, and then the model with the best ranking score for each complex was selected for visualization using the PyMOL Molecular Graphics System, v3.0 Schrödinger, LLC.

## Supplementary information

Supplementary figure

Supplementary table

## Data Availability

Data used in the preparation of this article were obtained from the Global Parkinson’s Genetics Program (GP2; https://gp2.org). Specifically, we used Tier 2 data from GP2 release 9 (10.5281/zenodo.14510099). GP2 data are available on AMP-PD (https://amp-pd.org).

## Code availability

All code generated for this article, and the identifiers for all software programs and packages used, are available on GitHub (https://github.com/GP2code/Multiancestry_LRRK2_p.A419V)and were given a persistent identifier via Zenodo (10.5281/zenodo.17701937). Genotyping imputation, quality control, ancestry prediction, and processing was performed using GenoTools v1.0, publicly available on GitHub (https://github.com/GP2code/GenoTools).
