# Supplementary material for: Association of LRRK2 p.A419V with Parkinson’s Disease in East Asians and analysis of age at onset
Source: NPJ Parkinsons Dis. 2026 Feb 2;12:51. doi: 10.1038/s41531-026-01265-3 (PMC12913921; doi:10.1038/s41531-026-01265-3)

Supplementary Figure 1: Cluster plot for *LRRK2* p.A419V on Neurobooster array (NBA)

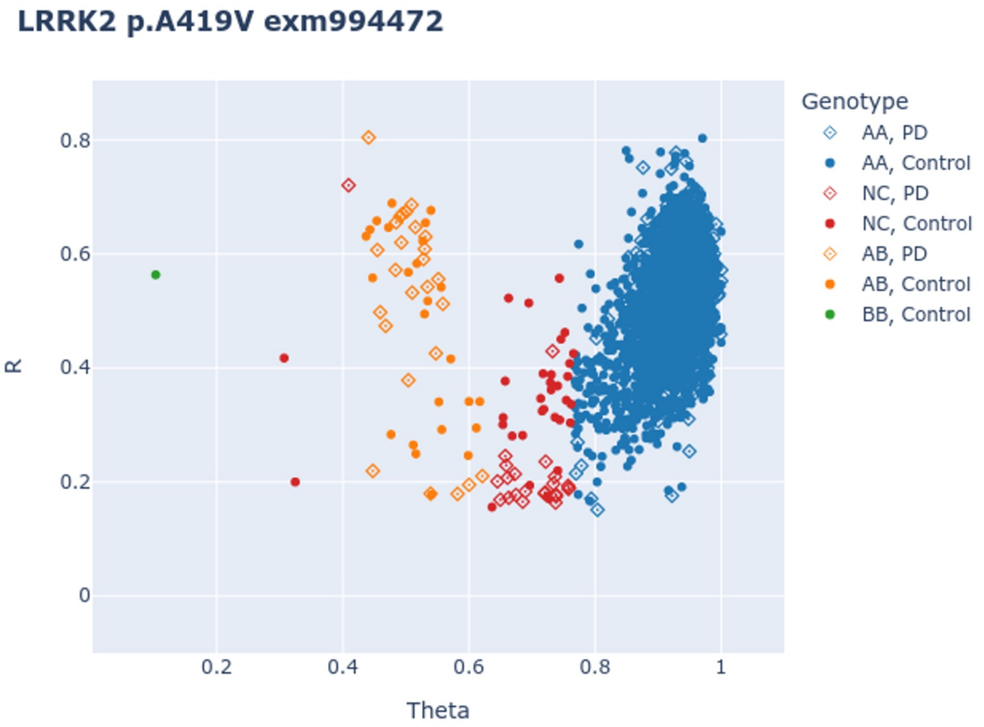

Abbreviation:
AA, homozygous reference individual
AB, heterozygous individual
BB, homozygous alternate individual
NC, no call (genotype failed)

Supplementary Figure 2: Scree Plot for GP2 release 9 data across ancestries

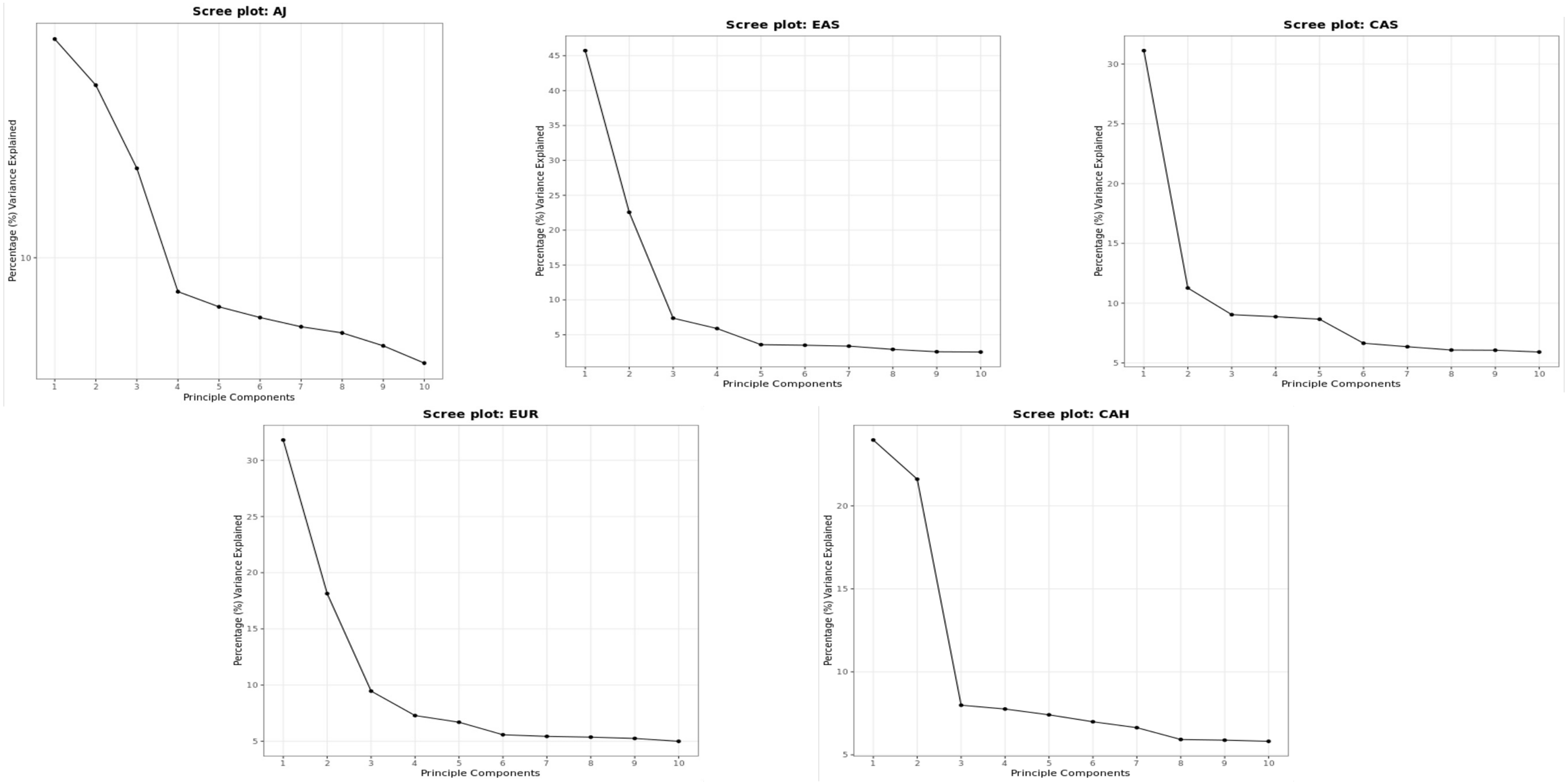

Supplementary Figure 3: Forest plot of effect size estimates (β) and 95% confidence intervals for the association between *LRRK2* p.A419V and Parkinson's disease age at onset across East Asian cohorts through fixed-effects meta-analysis

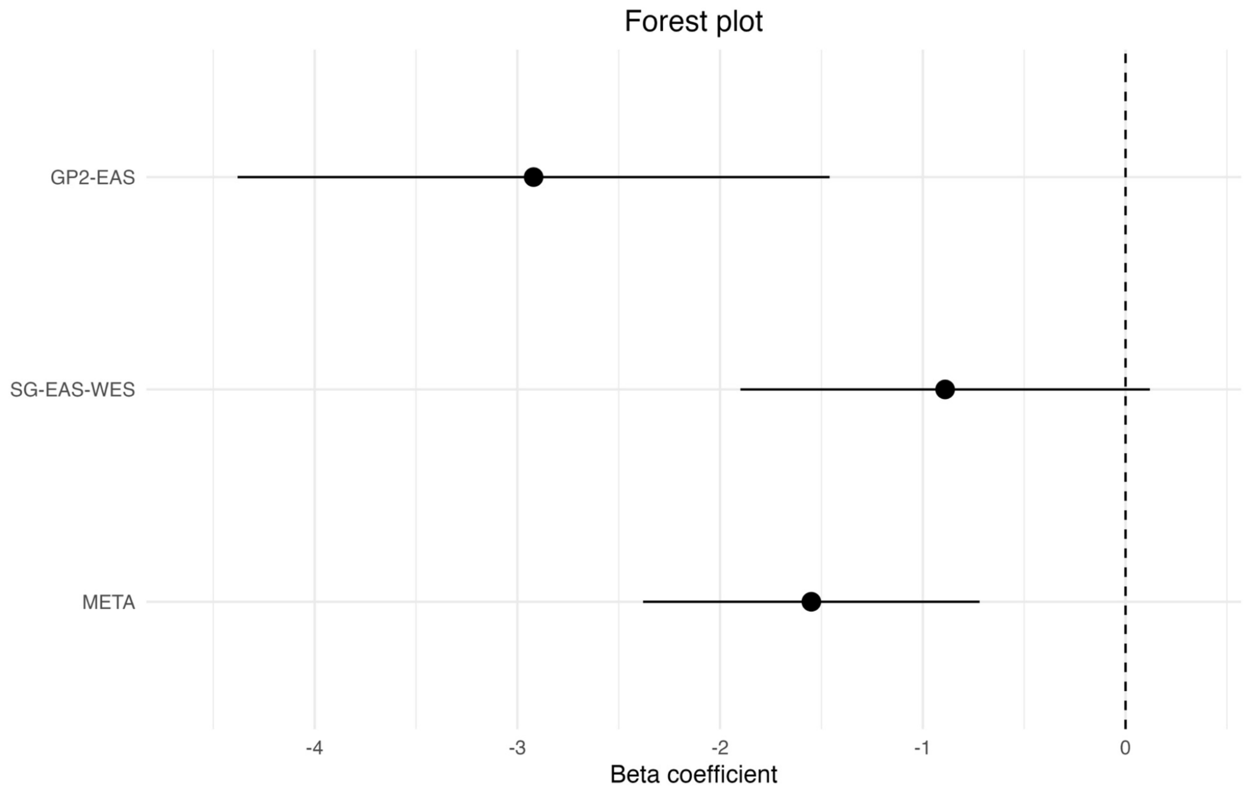

Supplementary Figure 4: Comparison of the Predicted LRRK2 Structure with Available X-ray Structure Regions. Structural comparison of the predicted (cyan) with the available X-ray (PDB: 7LHW) structures of LRRK2 highlights the structural similarity, with the RMSD value of 0.992 for Cα in the regions that are present in both structures. The predicted ARM domain, absent in the available X-ray structure, is shown in on the left with the A419V residue site (red). This comparison provides confidence in the reliability of the predicted structure for visualizing the ARM domain.

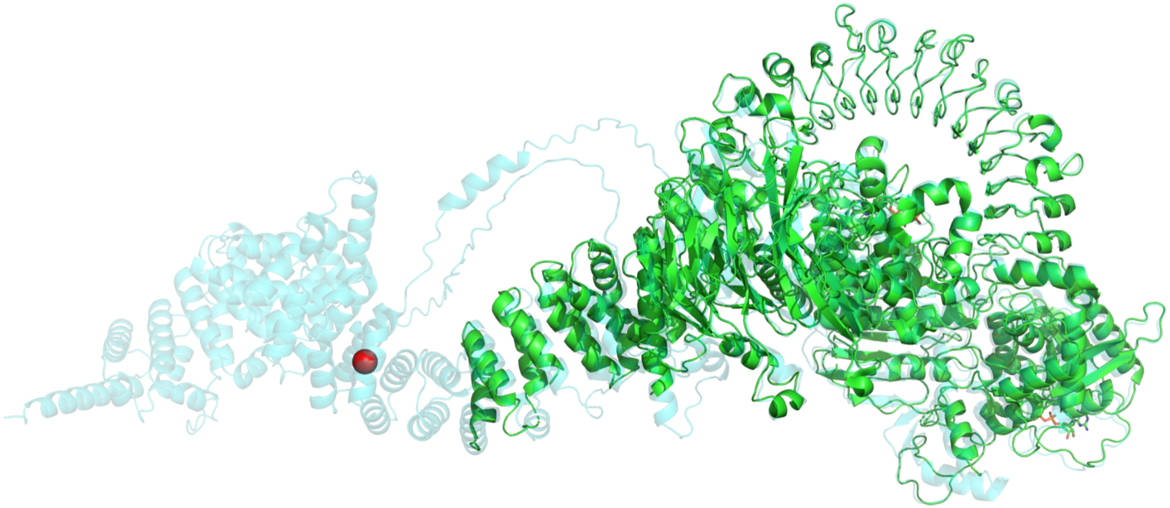

Supplement: Supplementary file 1 — Supplementary figure [file 41531_2026_1265_MOESM1_ESM.pdf]
